# Sex specific pattern of adipose expansion, inflammation and dysfunction with short term high fat diet exposure

**DOI:** 10.3389/fendo.2026.1814026

**Published:** 2026-06-23

**Authors:** Julia Skibniewska, Symmarana Desai, Lucy Cassell-Kelley, Jessica Maung, Abhimanyu Sastry, Janie McMillin, Heather Day, Kanakadurga Singer, Mita Varghese

**Affiliations:** Department of Pediatrics, Michigan Medicine, University of Michigan, Ann Arbor, MI, United States

**Keywords:** adipose, inflammation, insulin resisitance, Olink, sex differences

## Abstract

**Background:**

While body mass index (BMI) and weight gain are commonly used indicators of metabolic disease risk, intrinsic features of adipose tissue function such as adipocyte hypertrophy and inflammation may serve as earlier and more accurate predictors of metabolic dysfunction. Sex specific responses in adipose function may lead to sex differences in how adiposity associates with metabolic disease.

**Objective:**

This study aimed to determine the early sequence of events leading to adipose tissue dysfunction and metabolic inflammation in response to a high-fat diet (HFD), with a focus on sex-specific differences in adipose and immune responses during short-term high fat dietary exposure.

**Methods:**

Male and female C57BL/6J mice were fed either a normal chow diet or HFD (60% fat) for 1, 2, 4, 6 weeks, or for 16 weeks, starting at 6 weeks of age. Adipose tissue mass, adipocyte size, glucose and insulin levels, inflammatory gene expression, and adipose tissue macrophage (ATM) populations were measured using immunohistochemistry, flow cytometry, gene expression, and metabolic assays. Serum and GWAT explant media proteomic evaluations were conducted using the Olink platform.

**Results:**

HFD induced rapid adipose expansion in both sexes, but male mice exhibited greater weight gain, adipocyte hypertrophy, and insulin resistance over time. Males also demonstrated an earlier and more pronounced accumulation of pro-inflammatory CD11c^+^ ATMs in gonadal adipose tissue, alongside increased inflammatory gene expression and inflammatory chemokines and cytokines. Female mice exhibited a delayed and less robust inflammatory response. Notably, metabolic dysfunction, including hyperinsulinemia, preceded inflammation, particularly in males.

**Conclusion:**

Short term HFD induces sex-specific adipose tissue remodeling and metabolic dysfunction, with males showing earlier onset of both adipocyte hypertrophy and inflammation. These findings highlight the importance of sex as a biological variable in early metabolic disease development and suggest that insulin resistance may precede inflammation, which precedes metabolic dysfunction in the pathogenesis of adipose dysfunction.

## Introduction

Traditionally, weight and BMI (Body Mass Index) have been the standard indicators for assessing an individual’s risk of developing metabolic disease such as obesity and type 2 diabetes (1, 2). However, BMI is known to not be an accurate marker of metabolic disease risk (3). More than weight gain itself, intrinsic properties of an individual’s adipose tissue, such as adipocyte size and composition, as well as predisposition to inflammation, may serve as more accurate predictors of metabolic disease risk.

Adipose tissue expands through two primary mechanisms. Hyperplasia, where adipocytes multiply in number, and hypertrophy, where adipocytes increase in volume. Hypertrophy has been primarily associated with adipose tissue dysfunction (4). Dysfunctional adipose tissue is also characterized by inflammation, that drives metabolic impairment. This inflammation is largely mediated by adipose tissue macrophages (ATM), which shift their phenotype in response to environmental factors such as high fat diet (HFD), especially in the visceral adipose depot (5–7). Notably, this inflammation is not consistent across all individuals with higher body mass, which is why BMI is not an ideal marker for adipose function and metabolic disease (2, 8). For example, distribution of more visceral adipose tissue is linked to a higher cardiometabolic risk. Owing to sex differences in fat mass and distribution (9–11), pathogenesis of inflammation as a result of HFD is sexually dimorphic (12–14). This could explain sex differences in the prevalence of cardiovascular and other metabolic diseases, where premenopausal women exhibit a better lipid profile compared to men and postmenopausal women, as shown by lower levels of total cholesterol, low density lipoprotein (LDL), and triglycerides along with higher high-density lipoprotein (HDL) concentrations (15–17). Adipose tissue dysfunction and inflammation are both precursors to HFD-induced insulin resistance (18, 19). Yet, early in the response to HFD, it is unclear whether inflammation or adipose dysfunction occurs first. Understanding what changes occur early in the evolution of obesity may help us identify individuals at risk of cardiometabolic disease. Furthermore, assessing sex differences in early responses to HFD will help us determine more targeted preventative approaches to the development of metabolic disease.

In rodent obesity models, exposure to a prolonged (>12 weeks) HFD results in systemic and adipose tissue inflammation (19, 20). Other studies, focused in males, have shown that even a short-term exposure to HFD can be associated with inflammation and a shift in ATM phenotype (21–23), driving insulin resistance. With only 3 days of HFD feeding, mice showed systemic insulin resistance, glucose intolerance and increase in CD11c^+^ ATMs, monocyte chemoattractant protein (Mcp1) expression and CD68 (23). Another study showed that after 3 weeks on HFD, there were no significant changes in the expression of inflammatory markers such as macrophage antigen -1 (Mac-1), F4/80, Tnfα, Il6 and Il10 (24). A significant increase in these markers was only observed after a period of 16 weeks on HFD (24). Therefore, the duration of the experimental diet does have an impact on inflammation response.

While we know that sex-hormones directly drive local adipocyte responses to excess lipid-induced expansion in visceral vs subcutaneous depots (25–28), little is known about the sexually dimorphic responses to short term HFD. Estrogen may downregulate inflammation in adipose tissue through indirect regulation of hypoxia inducible factor (HIF-1), which normally promotes inflammation and fibrosis of adipose tissue (22). In chronic HFD conditions, sexually dimorphic inflammation differences are more systemic. Male monocytes exhibit increased migration in response to MCP1, a key inflammatory chemokine in adipose tissue (29, 30). In contrast, female monocytes showed migration but failed to further upregulate inflammatory cytokines in response to a dietary fatty acid, palmitate (29). This suggests that females may not react as robustly to high fat exposures (31). Ultimately these differences in monocyte recruitment add to the local inflammation and lead to males showing greater susceptibility to the accumulation and effects of pro-inflammatory ATMs.

Adipose tissue as the most expanded tissue depot in obesity is a major contributor to the insulin resistance seen in HFD exposure compared to non-adipose tissues (32). Specifically, visceral adipose tissue or gonadal white adipose tissue (GWAT) expansion in mice is linked with insulin resistance, while subcutaneous adiposity can maintain metabolic health (7, 33, 34). Understanding early responses in GWAT to HFD exposure can predict long term insulin resistance since adipose tissue becomes insulin resistant before other insulin-responsive tissues, such as skeletal muscle (24, 32). This development is relatively rapid in the adipose tissue, within the first three weeks on HFD (23) with excess lipid accumulation in non-adipose tissue being dependent on the storage capacity of the fat depots (24). There is ongoing debate over the sequence of these short-term effects of HFD. One study of an all-male mice cohort showed evidence of insulin resistance developing much earlier than inflammation (24). Given that the inflammatory response manifests differently in female mice, the sequence of these effects may vary in female cohorts. Furthermore, females have been shown to express higher levels of UCP-1 in their adipose tissue, as well as higher expression of genes related to adipose function (35). Insulin resistance is associated with adipose tissue dysfunction, making it crucial to determine the onset of resistance in exploring the relationship between inflammation and adipose tissue dysfunction.

Other short-term effects that result from HFD include liver fat accumulation and elevated lipid levels in mice. As early as one week on HFD, liver fat accumulation and elevated free fatty acid (FFA) levels were observed in the experimental mice contributing to hepatic insulin resistance (35). In the extreme short term, fat mass is significantly increased in HFD mice while overall body mass remains relatively unchanged (35). With chronic HFD, adipocyte lipolysis increases circulating FFAs but how much of this is determined by the early response to HFD is unknown. The chemokine TNFα also stimulates adipocyte lipolysis (35), suggesting that the inflammatory response associated with an excess fat diet could be contributing to these increased levels of FFAs. In omental tissue of obese humans, female adipose cells were smaller and exhibited a lower basal lipolysis rate than male adipose cells (36, 37). Moreover, women with increased adiposity have more efficient triglyceride fatty acid uptake than men (36). Hence, when evaluating comparisons during a short term HFD, it is crucial to consider both fat mass and body mass to yield statistically significant insights.

To understand if obesity outcomes such as inflammation changes or insulin resistance resulting in local adipocyte dysfunction precedes the other in males and females, we sought to conduct parallel short-term HFD experiments. We chose 1 week, 2 week, 4 week, 6 week and 16 weeks of HFD challenge. The rationale behind the shorter HFD challenge from 1 to 6 weeks was to investigate the origin of insulin resistance and inflammation. The longer time period of 16 weeks is an established HFD model of obesity that induces insulin resistance and inflammation in males (14, 38, 39). We hypothesized that in males, insulin resistance would precede inflammation and that males would exhibit insulin resistance as early as 1 week of HFD prior to an inflammatory response. For females on HFD, we hypothesized that insulin resistance and inflammation would require a longer dietary challenge.

## Methods

### Animal care and models

Male and female C57Bl/6J (WT) mice were used for this experiment. Animals were housed in a pathogen-free facility and fed varying diets. They were given free access to food and water. Mice were fed a high fat diet (HFD, 60% calories from fat) or normal diet (4.5% calories from fat) for 1, 2, 4, 6 and for 16 weeks HFD challenge starting at 6 weeks of age. **Supplementary Table 1** shows the total number of mice used across all experiment endpoints by sex, diet and the different diet timepoints of experimental analysis. Mice were sacrificed at the conclusion of their HFD periods. Control mice (ND) were sacrificed at the same time as their HFD counterparts. Serum and tissues were collected from the mice, including blood (via cardiac puncture), gonadal white adipose tissue (GWAT), inguinal white adipose tissue (IWAT), liver, and bones (bilateral femur and tibia). Mice were weighed immediately before they were sacrificed, and tissues (GWAT, IWAT, liver, pancreas) were weighed before collection. Animal protocols were in compliance with the Institute of Laboratory Animal Research Guide for the Care and Use of Laboratory Animals and approved by the University Committee on Use and Care of Animals at the University of Michigan (animal welfare assurance number: A3114-01).

### Metabolic assays

Metabolic testing included fed glucose and serum collection after 1, 2, 4 and 6 weeks of HFD.

### Flow cytometry

Adipose tissue fractionation and flow cytometry analysis were performed as described previously (38). Briefly, whole adipose tissue was minced and digested with type II collagenase (Sigma; 1 mg/ml in RPMI media) for 15 to 30 min at 37 °C on a rocker. Filtrated samples were spun at 500*g* for 10 min, and red blood cell lysis was conducted (Biosciences; 00-4333-57). Stromal vascular fraction cells were stained with anti-mouse CD45 eFluor450 (30-F11 monoclonal; Invitrogen), CD64 PE (X54-5/7.1 monoclonal; BD Pharmingen), and CD11c APC or eFluor 780 (N418 monoclonal; Invitrogen) (14). Gating was performed for macrophage populations and by CD45 gates to determine ATMs (14).

### Immunohistochemistry and immunofluorescence staining

Gonadal adipose tissues (GWAT) were formalin fixed, paraffin embedded, sectioned at 5 μm and immuno-stained with antibodies directed against Mac-2 (MAC-2; Galectin-3; eBioM3/38) for crown-like structure/macrophage staining, caveolin (CAV-1; Cell Signaling) for adipocytes, and DAPI for nuclear stain. Images were captured with an Olympus IX-81 fluorescent microscope (12, 39).

### Adipocyte sizing

For histology, tissues were formalin fixed, paraffin embedded, sectioned at 5 μm, and stained with H&E. H&E staining was performed by the University of Michigan’s Comprehensive Cancer Center Histology Core. Adipocyte sizing (14, 39) was conducted by capturing multiple TIFF-gray-scale images, and adipocyte circumference was determined with ImageJ software (the National Institutes of Health). Pixel areas of all individual cells in 3 to 5 areas were analyzed and averaged for each condition.

### Myeloid CFU

Femurs were extracted from the animals, and bone marrow (BM) was isolated from the femur and plated for myeloid colony assays as previously described (14). Briefly, BM cells from femurs were flushed with Iscove’s modified Dulbecco’s medium (IMDM), resuspended in MethoCult (Stem Cell Technologies) medium and plated (10,000 cells/plate) for granulocyte (G)/macrophage (M) assay. After 7 days, plates were scanned for CFU colonies. CFUs were further classified into granulocytes (G) and macrophages (M) based on size of the colonies (https://cdn.stemcell.com/media/files/manual/MA28405-Mouse_Colony_Forming_Unit_Assays_Using_MethoCult.pdf).

### Quantitative RT-PCR

RNA was extracted from adipose tissue using Trizol LS (Life Technologies) and cDNA was generated using a High-Capacity cDNA Reverse Transcription Kit (Applied Biosystems). SYBR Green PCR Master Mix (Applied Biosystems) and the StepOnePlus System (Applied Biosystems) were used for real-time quantitative PCR. *Arbp* expression was used as an internal control for data normalization. Samples were assayed in duplicate, and relative expression was determined using the 2-ΔΔ CT method. Primers used in the studies are provided in **Supplementary Table 2**.

### Olink proteomic analysis

Proteins in serum and GWAT explant media were measured using the Olink^®^ Target 96 Mouse Exploratory Panel* (Olink Proteomics AB, Uppsala, Sweden) according to the manufacturer’s instructions. This assay employed the Proximity Extension Assay (PEA) technique, documented in prior literature (40, 41), allowed for the concurrent analysis of 92 analytes with 1 µL of sample. Briefly, the assay employed two oligonucleotide-labeled antibody probes that specifically bind to their respective protein targets. Upon proximal binding of the probes to the target protein, the attached oligonucleotides undergo hybridization. This process is further amplified by the introduction of DNA polymerase, triggering a proximity-induced polymerization reaction that produced a unique PCR-readable DNA sequence. The amplified DNA is then quantified using a microfluidic real-time PCR instrument (Signature Q100). Following this, the data was normalized against an internal control to correct for variations within runs. Quantitative results are reported as Normalized Protein Expression (NPX) values, which are arbitrary units expressed on a log2-scale that directly correlates with the protein expression levels, where higher NPX values indicated greater protein concentrations.

The assay was conducted on serum from 4 weeks, and 16 weeks HFD-fed male and female mice. The assay was also conducted on conditioned media samples of GWAT explants from 4 weeks and 6 weeks HFD-fed male and female mice. The assay was performed at the Immuno-monitoring laboratory (Cleveland Clinic, OH).

### Statistics

All statistical analyses were conducted in GraphPad Prism (v10.4.1). For comparisons involving sex and diet, two-way analysis of variance (ANOVA) was used to test main and interaction effects. *Post hoc* pairwise comparisons were performed using multiple comparison test using Sidak’s correction. Statistical significance was defined as FDR or p.adj < 0.05 unless otherwise stated. All values are presented as mean ± SEM. Linear correlation with simple regression analysis was used for correlation analysis. For two-group comparisons in MetaboAnalyst 6.0, univariate analysis with the Student’s t test and variable fold change analysis with an FDR of 0.05 was conducted. Differences with a P ≤ 0.05 were considered significant. PCA analysis was performed in MetaboAnalyst with permutational analysis of variance (PERMANOVA) to compare groups of multivariate data. Heatmap analysis was performed with p values from t-tests of NPX values (from Olink analysis) with the euclidean distance metric. Volcano plots were created to visualize significant proteins satisfying both FDR (<0.05) and fold change (>2). Bar graphs were analyzed by 2-way ANOVA interaction accounting for main effects of sex and diet followed by *post hoc* analysis for multiple comparisons corrected with Sidak’s method in GraphPad Prism (v10.4.1).

## Results

### Short term HFD enhances adiposity in both male and female mice

**Figure 1:**
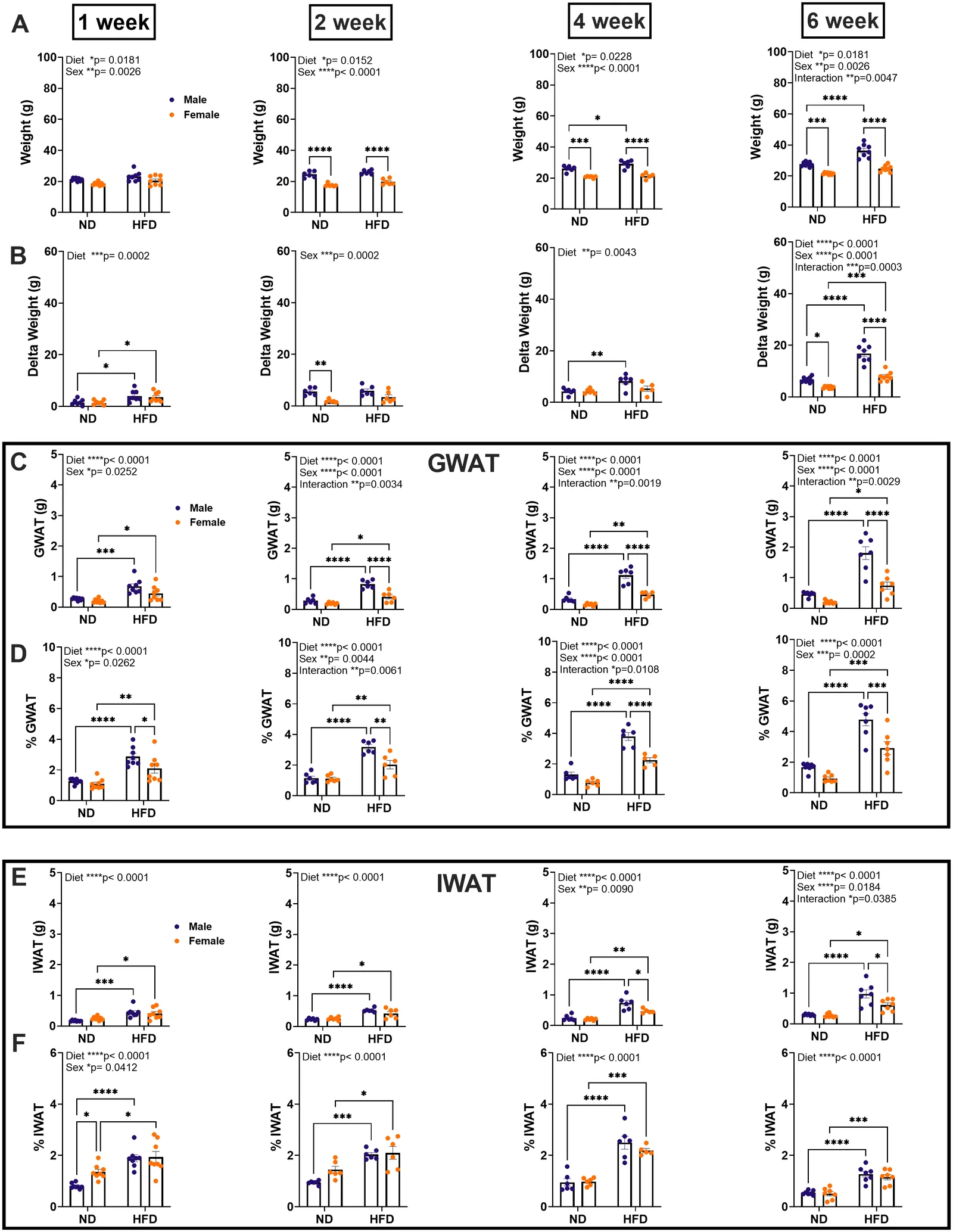
Sex differences in total body adiposity and adipose tissue weights in response to short term and long term HFD. **(A)** Total body weights at 1 week, 2-week, 4 week and 6 weeks of ND and HFD. **(B)** Delta weight at 1 week, 2-week, 4 week and 6-week ND and HFD. For **(A, B)** N = 1 week M ND (8), M HFD (8), F ND (8) and F HFD (8); N = 2 week M ND (6), M HFD (6), F ND (6) and F HFD (6); N = 4 week M ND (6), M HFD (6), F ND (6) and F HFD (5); N = 6 week M ND (8), M HFD (8), F ND (8) and F HFD (8). **(C)** Total GWAT weight at 1 week, 2-week, 4 week and 6-week ND and HFD. **(D)** % GWAT weight at 1 week, 2-week, 4 week and 6-week ND and HFD. **(E)** Total IWAT weight at 1 week, 2-week, 4 week and 6-week ND and HFD. **(F)** % IWAT weight at 1 week, 2-week, 4 week and 6-week ND and HFD. For (C - F) N = 1 week M ND (8), M HFD (8), F ND (8) and F HFD (8); N = 2 week M ND (6), M HFD (6), F ND (6) and F HFD (6); N = 4 week M ND (6), M HFD (6), F ND (6) and F HFD (5); N = 6 week M ND (7), M HFD (7), F ND (7) and F HFD (7). Data analysis was performed by 2-way ANOVA accounting for sex and diet followed by *post hoc* analysis for multiple comparisons corrected with Sidak’s method. Data shown as average ± SEM. ∗p < 0.05, ∗∗p < 0.01, ∗∗∗p < 0.001, and ∗∗∗∗p < 0.0001. Statistics from diet and sex interaction are shown.

Animals were assessed after 1, 2, 4, or 6 weeks of dietary challenge for changes in body and adipose tissue weights. We have earlier shown that with 16 weeks of HFD, males gained more body weight than females (14, 39). At 1 week of HFD, neither male nor female animals showed significant changes in body weight compared to their ND and HFD counterparts (**Figure 1A**). Following 2 weeks of HFD, males gained significantly more weights compared to females on either diet and continued to do so into the 4 week and 6 week HFD challenge (**Figure 1A**). With short term HFD exposure, male mice on HFD were noted to be heavier than their ND male counterparts at 6 weeks after HFD (**Figure 1A**; sex x diet interaction p<0.05). When assessing the change in weight (delta weight) from baseline, diet had a significant impact as early as 1 week and then at 4 weeks (**Figure 1B**). At 6 weeks of diet challenge, delta weight showed that both sexes were significantly heavier than their ND counterparts and males on either diet weighed more than females (**Figure 1B**; sex x diet interaction p<0.05).

Next, we assessed changes in GWAT and IWAT weights with short-term HFD exposure. Across all time points from 1 week, 2-week, 4 week and 6 week, GWAT mass was significantly increased in the HFD groups compared to the ND groups in both sexes (**Figure 1C**). However, at 2-week, 4 week and 6 weeks, GWAT weights significantly increased in HFD males than females (**Figure 1C**; sex x diet interaction p<0.05). Increase in % GWAT was observed in both males and females on HFD as early as 1 week of the dietary challenge (**Figure 1D**). Further increases in % GWAT were observed in HFD males than HFD females after 2 weeks of HFD and continuing similarly through 4 weeks of HFD (**Figure 1D**; sex x diet interaction p<0.05). At 6 weeks, HFD mice showed higher % GWAT weights than ND mice in both sexes (**Figure 1D**). IWAT weight was significantly increased in HFD male and female mice compared to their ND counterparts at 1 week, 2-week, 4 week and 6 weeks of HFD (**Figure 1E**). After 4 weeks, male and female HFD mice showed larger increases in IWAT weight, compared to their ND controls (**Figure 1E**). This increase in IWAT weight in HFD males was consistently higher than ND and HFD females at 6 weeks (**Figure 1D**; sex x diet interaction p<0.05). While the % IWAT weight was higher in ND females compared to males at the early time point of I week, this however normalized by 6 weeks of HFD (**Figure 1F**). Overall, % IWAT showed the same pattern as % GWAT, with HFD mice having higher % IWAT than ND mice in both sexes across all HFD exposure timepoints (**Figure 1F**).

### Male animals have increased insulin secretion even in short term HFD challenges compared to females

**Figure 2:**
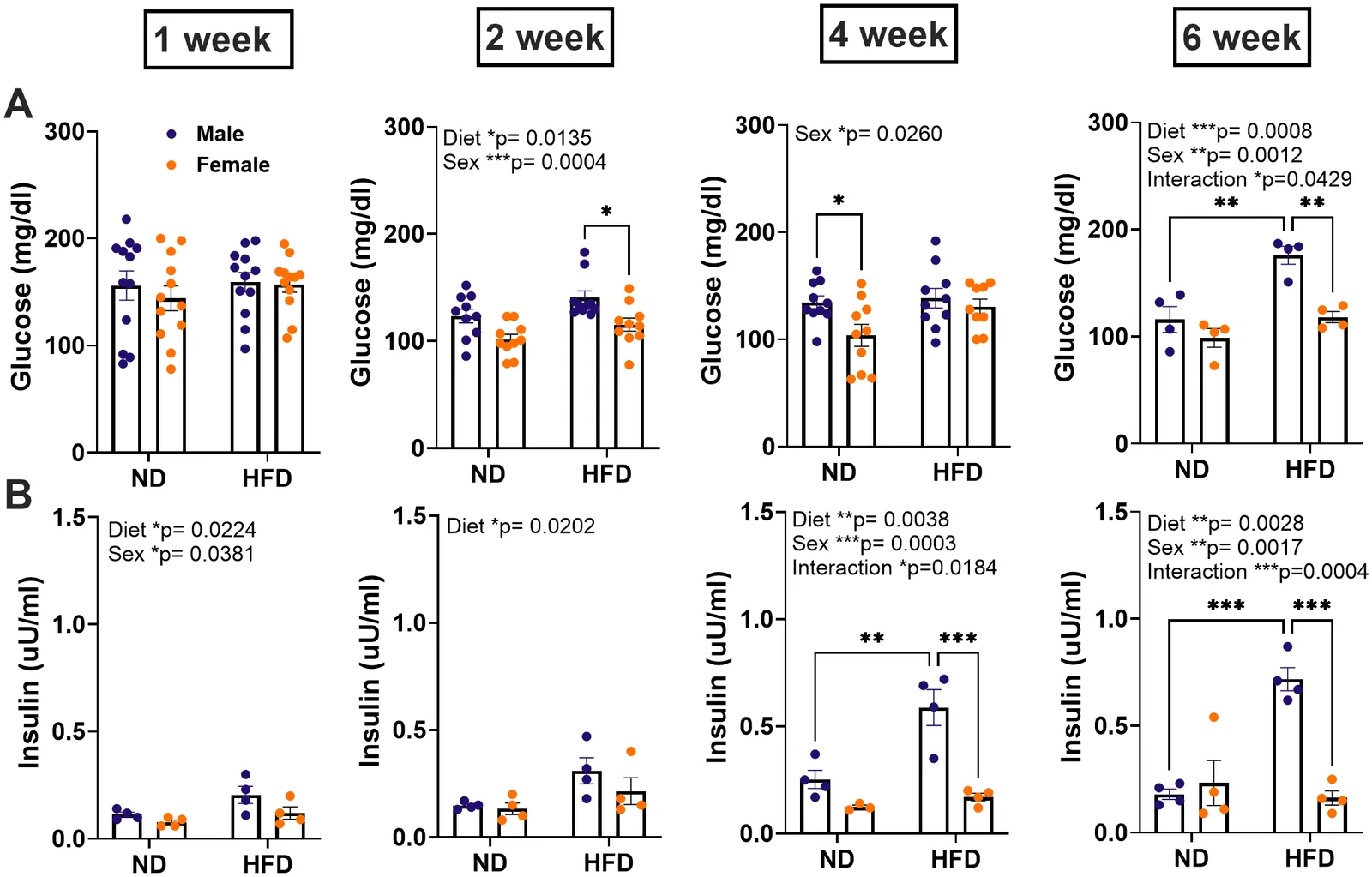
Insulin sensitivity is higher in females than males with short term HFD. Glucose levels in male and female serum with **(A)** 1 week, 2-week, 4 week and 6-week ND and HFD. N = 1 week M ND (12), M HFD (12), F ND (12) and F HFD (12); N = 2 week M ND (10), M HFD (10), F ND (10) and F HFD (10); N = 4 week M ND (10), M HFD (10), F ND (10) and F HFD (9); N = 6 week M ND (4), M HFD (4), F ND (4) and F HFD (4). Insulin levels in male and female serum with **(B)** 1 week, 2-week, 4 week and 6-week ND and HFD. N = 1 week M ND (4), M HFD (4), F ND (4) and F HFD (4); N = 2 week M ND (4), M HFD (4), F ND (4) and F HFD (4); N = 4 week M ND (4), M HFD (4), F ND (4) and F HFD (4); N = 6 week M ND (4), M HFD (4), F ND (4) and F HFD (4). Data analysis was performed by 2-way ANOVA accounting for sex and diet followed by *post hoc* analysis for multiple comparisons corrected with Sidak’s method. Data shown as average ± SEM. ∗p < 0.05, ∗∗p < 0.01, ∗∗∗p < 0.001, and ∗∗∗∗p < 0.0001. Statistics from diet and sex interaction are shown.

To investigate changes in fed glucose levels and insulin secretion during the time course of HFD exposure, serum was assessed at the end of 1, 2, 4 and 6 weeks of HFD challenge from males and females. At 1 week of HFD, no significant changes in glucose levels were observed by sex or diet (**Figure 2A**). As early as 2 weeks on HFD, changes in glucose levels were observed by sex and diet, that persisted at 6 weeks of HFD (**Figure 2A**). At 4 weeks of HFD, glucose levels were significantly higher in ND males than females with a significant difference by sex (**Figure 2A**). At 6 weeks, males on HFD showed significantly higher glucose levels than ND males (**Figure 2A**). Even at 6 weeks of HFD, females did not show any significant changes in glucose levels compared to their ND counterparts and remained significantly lower than HFD males (**Figure 2A**; sex x diet interaction p<0.05). Next, when we assessed insulin levels, males on HFD had higher insulin levels compared to ND counterparts or HFD females, starting at 4 weeks of HFD challenge (**Figure 2B**; sex x diet interaction p<0.05). Insulin secretion was also significantly higher in HFD males compared to HFD females and ND males at 6 weeks of HFD exposure (**Figure 2B**; sex x diet interaction p<0.05).

#### Meta-inflammation is strongest in long term HFD fed male GWAT

**Figure 3:**
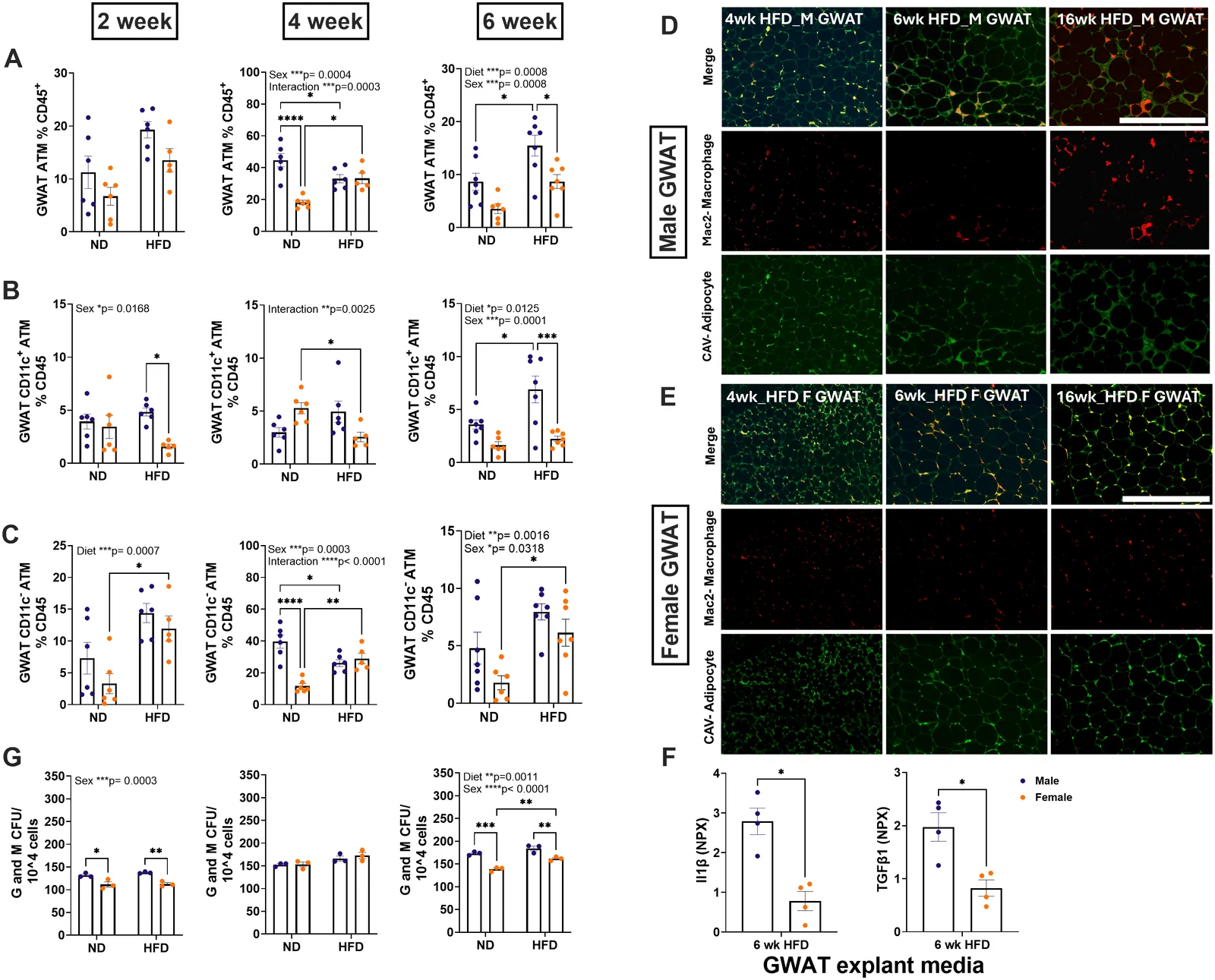
Short term HFD promotes pro-inflammatory ATMs in male mice GWAT. Flow cytometry quantitation as % of CD45 of **(A)** 2-week, 4 week and 6 week male and female ND and HFD total GWAT ATMs. Quantitation as % of CD45 of **(B)** 2-week, 4 week and 6 week male and female ND and HFD GWAT CD11c^+^ATMs. Quantitation as % of CD45 of **(C)** 2-week, 4 week and 6 week male and female ND and HFD GWAT CD11c^-^ATMs. For **(A–C)** N = 2 week M ND (6), 2 week M HFD (6), 2 week F ND (6), 2 week F HFD (5), 4 week M ND (6), 4 week M HFD (6), 4 week F ND (6), 4 week F HFD (5), 6 week M ND (7), 6 week M HFD (7), 6 week F ND (6) and 6 week F HFD (7). Representative immunofluorescence images of 4-week, 6 week and 16-week HFD **(D)** Male GWAT and **(E)** Female GWAT depicting Mac-2 labeling of CLS (red) and CAV labeling of adipocytes (green). Scale bar = 500 μm. **(F)** Olink analysis of IL1β and TGFβ1 in GWAT explant media from 6-week HFD male and females. N = 4/group. **(G)** Myeloid methylcellulose colony-forming units (CFUs) were counted and assessments were made for granulocyte-G and macrophage-M in 2-week, 4 week and 6-week HFD myeloid cells. N = 3/group. For **(A–C, G)**, data analysis was performed by 2-way ANOVA accounting for sex and diet followed by *post hoc* analysis for multiple comparisons corrected with Sidak’s method. For **(F)** data analysis was performed with unpaired t-tests. Data shown as average ± SEM. ∗p < 0.05, ∗∗p < 0.01, ∗∗∗p < 0.001, and ∗∗∗∗p < 0.0001. Statistics from diet and sex interaction are shown.

Chronic HFD exposure recruits circulating monocytes to sites of stress such as the adipose tissue to form pro-inflammatory macrophages often around re-modeling adipocytes in crown-like structures (CLS) (21, 42). To investigate changes in adipose inflammation with short term HFD exposure, we examined crown-like structures (CLS) through fluorescence imaging and assessed ATM populations (as a proportion of CD45^+^ leukocytes) through flow cytometry. During the 2 weeks of HFD, no significant differences were observed in total GWAT ATMs between ND and HFD groups of either sex (**Figure 3A**; left). At 4 weeks of HFD, female HFD GWAT ATMs increased significantly compared to female ND, while male HFD decreased compared to their ND counterparts (**Figure 3A**; main effect of sex and sex x diet interaction p<0.05; middle). There was an increase in GWAT ATMs by diet and showed a sex difference with more ATMs in HFD males compared to HFD females at 6 weeks (**Figure 3A**; right). There was a significant difference by sex in CD11c^+^ ATMs at 2 weeks of HFD where HFD males showed a significant elevation in CD11c^+^ ATMs compared to HFD females (**Figure 3B**; main effect of sex p<0.05; left) and remained persistently high at 6 weeks of HFD compared to females (**Figure 3B**; main effect of sex and diet p<0.05; right). There was also a significant increase in CD11c^+^ ATMs in HFD males compared to ND males at the 6-week timepoint (**Figure 3B**; main effect of sex and diet p<0.05; right). Females on 2, 4 and 6 weeks HFD did not show a significant increase in CD11c^+^ATMs compared to ND females (**Figure 3B**). However, as early as 2 weeks of HFD,females showed a significant increase in CD11c^-^ ATMs (**Figure 3C**; main effect of diet p<0.05; left) and remained consistently higher at 4 weeks (**Figure 3C**; middle) and 6 weeks of HFD (**Figure 3C**; right). At 4 weeks, ND males showed higher CD11c^-^ ATMs than ND females and HFD males (**Figure 3C**; sex x diet interaction p<0.05; middle).

Immunohistochemistry of GWAT sections showed no crown-like structures (CLS), but Mac-2 staining showed individual macrophages at 4 week HFD in both male (**Figure 3D**) and female GWAT (**Figure 3E**). Imaging also demonstrated an increased adipocyte size in male GWAT. At 6 weeks of HFD, an emergence of few CLS were observed only in male GWAT (**Figure 3D**) but not in female GWAT (**Figure 3E**). However, at 16 weeks HFD, male and female GWAT showed CLS with many more in males than females (**Figures 3D, E**). At 6 weeks of HFD, it became most apparent that male GWAT showed significantly more ATMs than female GWAT (**Figures 3D, E**). Consistent with this, GWAT explant derived media from 6 weeks of HFD demonstrated increased IL1β and TGFβ1 protein in media from male HFD compared to females (**Figure 3F**) by Olink analysis.

Given our prior studies demonstrating expansion of myelopoiesis in chronic HFD (14, 38), we further performed colony-forming unit assays with bone marrow to assess the changes in granulocyte and macrophage potential at shorter durations of HFD. Myeloid colonies (granulocyte (G) and macrophage (M)) were counted as colony-forming units (CFUs). Males of both diet groups had more CFUs than their respective female counterparts at 2 and 6 weeks of HFD (**Figure 3G**; left). There were no significant differences in CFUs at 4 weeks (**Figure 3G**; middle). Females showed a significant but subtle elevation in myeloid colonies numbers at 6 weeks of HFD challenge compared to ND females (**Figure 3G**; right). Overall, changes in BM myelopoiesis were not robustly altered in short term HFD as observed in chronic HFD (14).

### Pro-inflammatory gene expression is most upregulated in males on HFD with short term and long term HFD exposure

**Figure 4:**
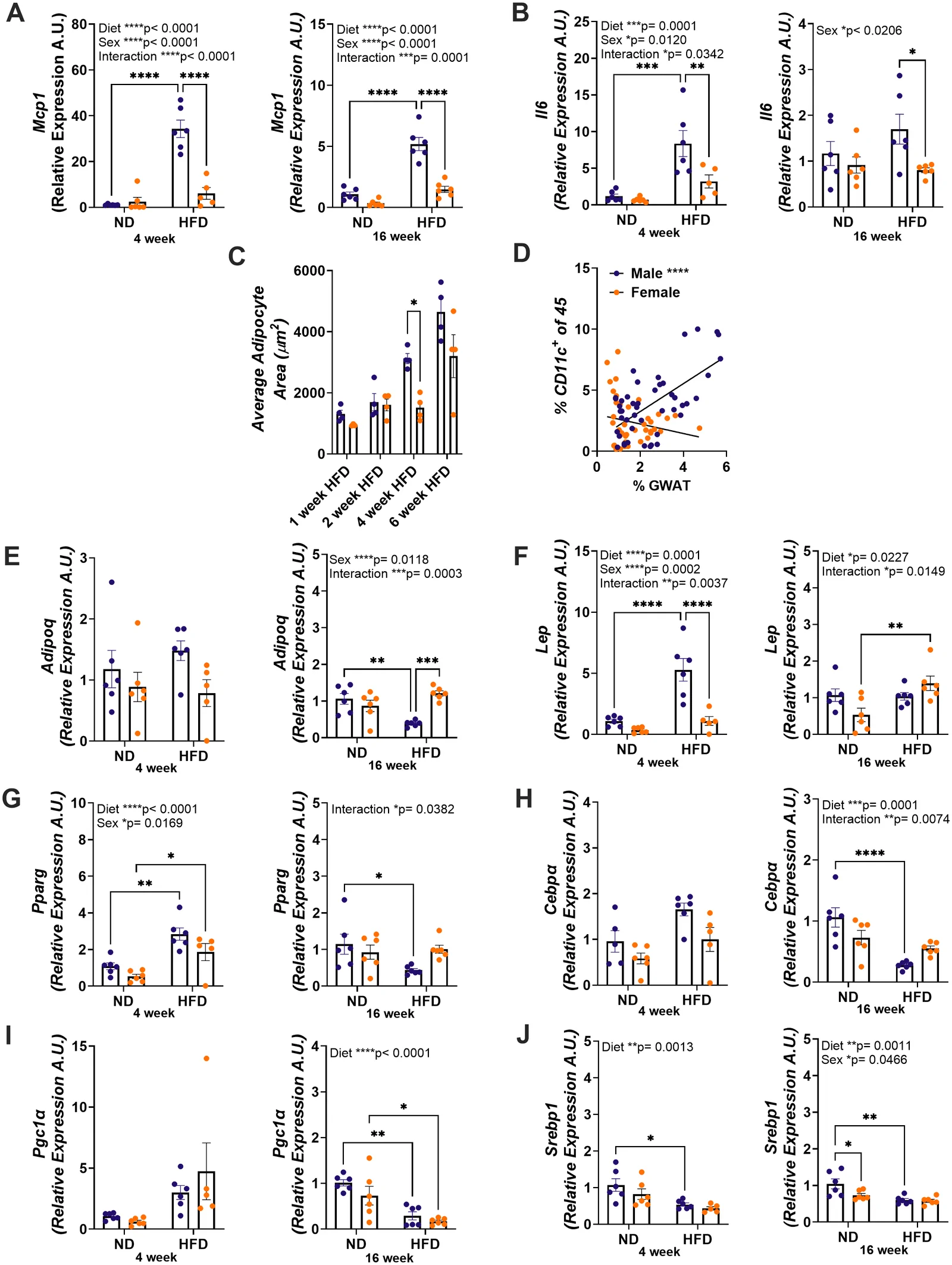
Short term HFD upregulates pro-inflammatory genes, induces adipocyte hypertrophy in male GWAT and correlates positively with increase in CD1lc+ macrophages, while long term HFD downregulated adipogenic genes. Expression of GWAT inflammation genes in 4 week and 16 week male and female HFD GWAT- **(A)** *Mcp1* **(B)** *Il6*. N = 4-week M ND (6), 4-week M HFD (6), 4-week F ND (6), 4 week F HFD (5), 16 week M ND (6), 16 week M HFD (6), 16 week F ND (6), 16 week F HFD (6). **(C)** Average adipocyte area from 1 week, 2-week, 4 week and 6-week HFD male and female GWAT. Unpaired t-test with Welch’s correction and *post-hoc* analysis for multiple comparisons by FDR method was performed. N = 4/group. **(D)** Simple linear regression plot of male and female % GWAT weight and GWAT CD11c^+^ ATMs from 1 week, 2-week, 4 week and 6-week ND and HFD. N = 1 week M ND (4), 1 week M HFD (4), 1 week F ND (4), 1 week F HFD (4), 2 week M ND (6), 2 week M HFD (6), 2 week F ND (6), 2 week F HFD (5), 4 week M ND (6), 4 week M HFD (6), 4 week F ND (6), 4 week F HFD (5), 6 week M ND (7), 6 week M HFD (7), 6 week F ND (6) and 6 week F HFD (7). R^2^ = 0.398 and ****p<0.0001 for male GWAT. Expression of adipogenic genes in 4 week and 16 week male and female HFD GWAT **(E)** *Adipoq* **(F)** *Lep* **(G)** *Pparg* **(H)** *Cebpa* **(I)** *Pgc1a* **(J)** *Srebp1*. A.U., arbitrary units normalized to *Arbp*. N = 4-week M ND (6), 4-week M HFD (6), 4-week F ND (6), 4 week F HFD (5), 16 week M ND (6), 16 week M HFD (6), 16 week F ND (6), 16 week F HFD (6). For **(A, B, E–J)**, data analysis was performed by 2-way ANOVA accounting for sex and diet followed by *post hoc* analysis for multiple comparisons corrected with Sidak’s method. Data shown as average ± SEM. ∗p < 0.05, ∗∗p < 0.01, ∗∗∗p < 0.001, and ∗∗∗∗p < 0.0001. Statistics from diet and sex interaction are shown.

Next, we investigated the effects of HFD on inflammation in the GWAT through gene expression studies after 4 and 16 weeks of HFD by qPCR. Looking first at inflammation, monocyte chemoattractant protein-1 (*Mcp1*) and interleukin 6 (*Il6*) expression as indicators of a cellular inflammatory response were assessed (30, 43). At 4 weeks of HFD, *Mcp1* expression was significantly upregulated in male HFD adipose tissue than HFD females and ND males (**Figure 4A**; sex x diet interaction p<0.05). This similar pattern was observed at 16 weeks of HFD (**Figure 4A**; sex x diet interaction p<0.05). *Il6* expression was also significantly upregulated in males on HFD after 4 weeks when compared with ND males and HFD females (**Figure 4A**; sex x diet interaction p<0.05). Unlike, *Mcp1* expression, after 16 weeks, *Il6* expression between HFD and ND males was no longer significant but showed a sex difference with HFD males having greater expression than females (**Figure 4B**; main effect of sex p<0.05). Thus, an increased inflammatory tone persisted in the 16-week HFD-fed male GWAT than female.

### Adipocyte hypertrophy in male GWAT begins earlier with 4 weeks of HFD exposure and correlates positively with increase in CD11c^+^ ATMs

With HFD exposure, lipid accumulation in adipose tissue triggers adipocyte hypertrophy and potentially driving myeloid inflammation (44) as seen in gene expression data (**Figures 4A, B**). To investigate changes in adipocyte hypertrophy with 1-, 2-, 4- and 6-week HFD exposure, images from H&E stained GWAT sections were assessed to determine average adipocyte size. Adipocyte size was significantly greater in male HFD GWAT adipocytes than females at 4 weeks of HFD (**Figure 4C**). Although a similar pattern was observed at 1, 2 and 6 weeks of HFD, the results were not significantly different (**Figure 4C**). With long term HFD exposure, prior results from our lab with a 16 week HFD showed that GWAT adipocyte average size and degree of adipocyte hypertrophy was similar between sexes (14). Consistent with sex differences in inflammation generation in response to adipocyte hypertrophy, we observed a sexually dimorphic relationship between GWAT weight increase and higher ATMs. Upon examination, a positive correlation was observed between male %GWAT weight and % CD11c^+^ ATMs which was not observed in female GWAT (**Figure 4D**).

### Genes regulating adipogenesis are altered in both sexes in the short term, but adipogenic activity diminishes in male GWAT after prolonged exposure to HFD

Adipose tissue expansion is driven either by the increase in adipocyte size (hypertrophy) or by the formation of new adipocytes from precursor differentiation in the process of adipogenesis (hyperplasia) (45). To determine adipogenic changes in GWAT with HFD exposure, we examined differences in regulation of adipogenesis by assessing expression of adiponectin (*Adipoq*), Leptin (*Lep*), peroxisome proliferator-activated receptor gamma (*Pparg*), CAAT enhancer-binding protein alpha (*Cebpa*), Peroxisome proliferator-activated receptor gamma coactivator 1-alpha (*Pgc1a*), and sterol regulatory element-binding protein 1 (*Srebp1*) genes. There were no significant differences in GWAT *Adipoq* expression after 4 weeks of HFD between males or females (**Figure 4E**). After 16 weeks, *Adipoq* expression in HFD males was significantly downregulated when compared with ND male GWAT (**Figure 4E**). However, after 16 weeks, *Adipoq* expression in HFD females was upregulated when compared with HFD males (**Figure 4E**; sex x diet interaction p<0.05). *Lep* expression was higher in HFD males than HFD females and ND males after just 4 weeks (**Figure 4F**; sex x diet interaction p<0.05). After 16 weeks, *Lep* expression was significantly upregulated in HFD females compared with ND females (**Figure 4F**). At 4 weeks of HFD, *Pparg* expression was higher in both male and female HFD GWAT than their ND counterparts (**Figure 4G**). After 16 weeks of HFD, *Pparg* expression was lower in HFD male GWAT compared to ND male GWAT (**Figure 4G**; sex x diet interaction p<0.05). *Cebpa* expression only changed at 16 weeks with lower expression in HFD male GWAT than ND males (**Figure 4H**; sex x diet interaction p<0.05). Similarly, *Pgc1a* also only changed at 16 weeks with expression being lower in HFD animals compared to ND in both sexes (**Figure 4I**). After 4 weeks, *Srebp1* expression was lower in HFD males than ND males (**Figure 4J**). After 16 weeks, *Srebp1* expression was still significantly lower in HFD males compared to ND male GWAT and lower in ND females respectively (**Figure 4J**).

### Protein analysis with Olink demonstrated a rise in circulating pro-inflammatory proteins at short-term HFD in males

**Figure 5:**
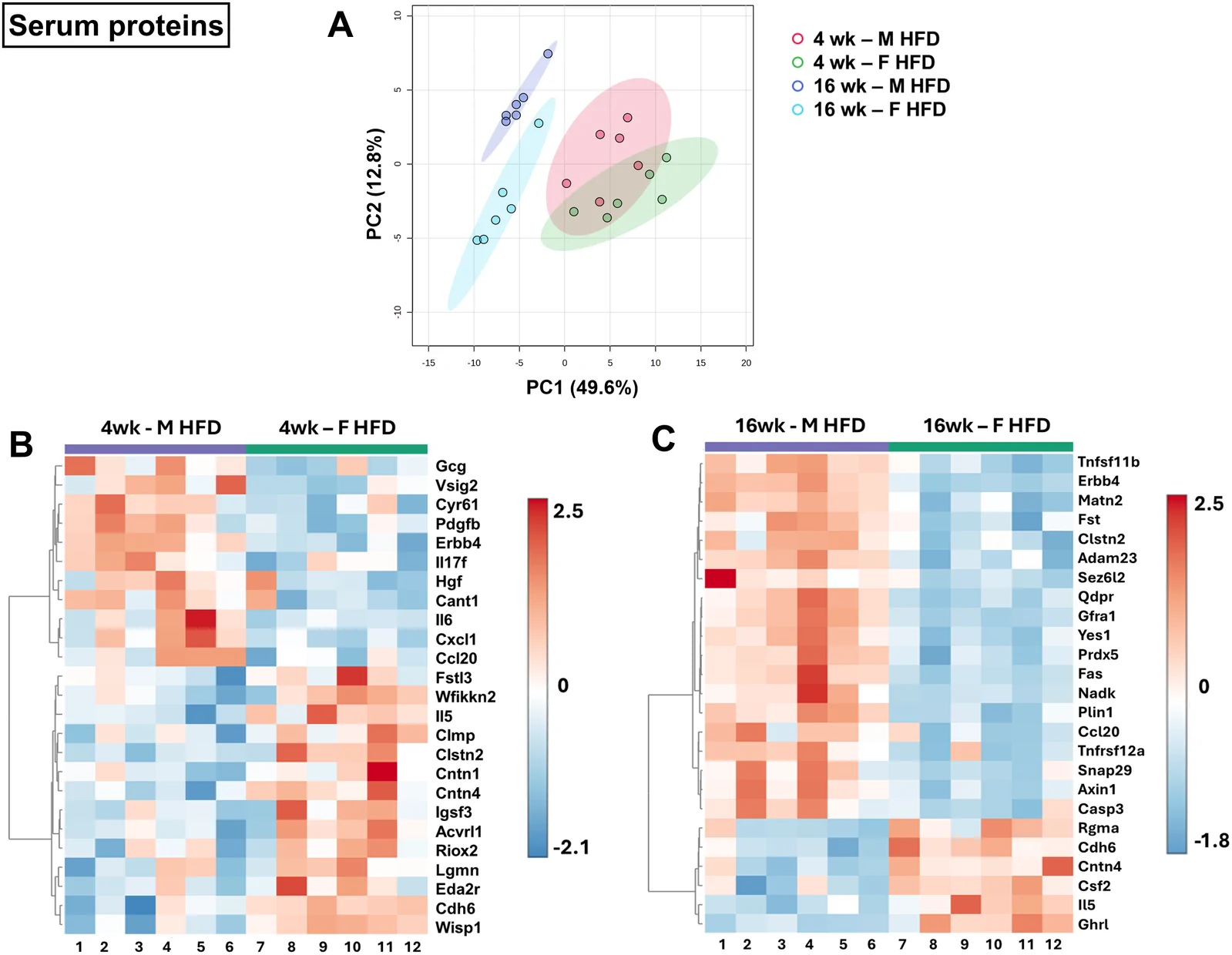
Olink analysis of 4 week and 16 week male and female HFD serum proteins. **(A)** Principal component analysis (PCA) plot of 4 week and 16 week male and female HFD serum samples. The statistical significances of the group patterns were evaluated in MetaboAnalyst with PERMANOVA. Distributions were computed using the Euclidean distance based on the PCs in the current display. Overall significance: F-value= 27.6; R-squared= 0.80545; p-value (based on 999 permutations) = 0.001. Heatmaps generated by MetaboAnalyst of top 25 upregulated or downregulated proteins ranked by t-test, in **(B)** 4 week and **(C)** 16 week male and female HFD serum samples. Top 25 proteins within the Olink^®^ Target 96 Mouse Exploratory Panel (**Supplementary Tables 3, 4**) were generated from p values of t-tests within 4 week and 6-week groups. N = 4-week M HFD (6), 4-week F HFD (6), 16-week M HFD (6), 16-week F HFD (6).

**Figure 6:**
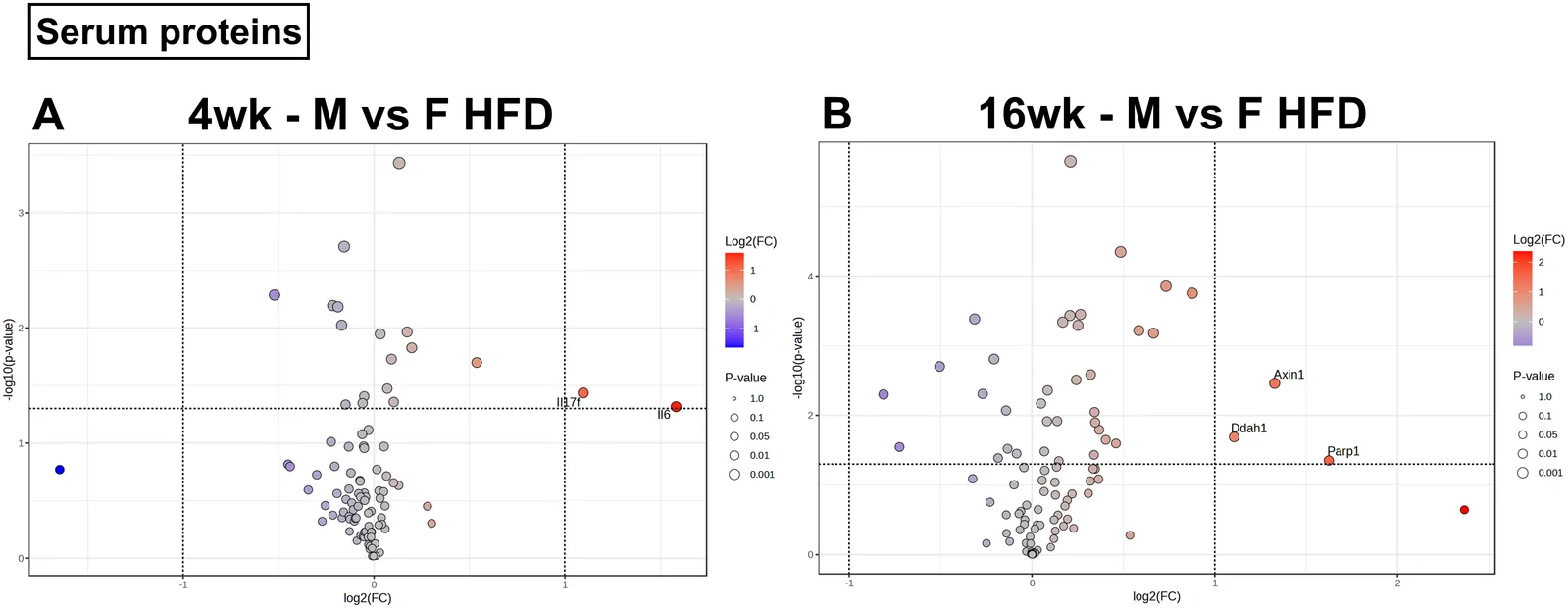
Olink proteins upregulated or downregulated with 4 week and 16-week HFD in male and female serum. Volcano plots depicting log2 fold changes in proteins (related to immune regulation, inflammation, cell migration, metabolic pathways, and tumor biology) and the corresponding significance values displayed as -log10 (p value) for **(A)** 4 week - M vs F HFD **(B)** 16 week - M vs F HFD. Each dot represents a protein, and the dot size and color intensity indicate significance. Red dots represent significantly upregulated and blue dots represent significantly downregulated proteins. Only proteins with p < 0.05 and fold change >2 are displayed. N = 4-week M HFD (6), 4-week F HFD (6), 16-week M HFD (6), 16-week F HFD (6).

To further investigate the origin of inflammation in our HFD timeline, we performed O-link analysis of circulatory proteins. This assay measured 92 proteins involved in a broad range of biological processes including immune regulation, inflammation, cell migration, metabolic pathways, and tumor biology (**Supplementary Tables 3, 4**). The assay was conducted on serum from 4 week and 16 weeks of HFD male and female mice. Principal component analysis (PCA) by PERMANOVA showed a distinct separation or greater variance between 16-week HFD male and 16-week HFD female serum samples, while 4 week HFD serum shared similarities between male and female sample groups (**Figure 5A**). Pair wise PERMANOVA plots and scores are shown in **Supplementary Figures 1A, B**. Heat map analysis shows the top 25 proteins (from p-values of t-tests of protein values) that were upregulated or downregulated between males and females in the 4 week (**Figure 5B**) and 16-week HFD serum samples (**Figure 5C**). Consistent with the PCA analysis, heatmaps showed distinct changes between male and female HFD serum proteins at both 4 weeks (**Figure 5B**) and 16 weeks of HFD (**Figure 5C**). To further understand the sex differences in specific protein changes between the short term 4-week HFD exposure and 16-week long term exposure, we generated volcano plots (**Figures 6A, B**). We performed comparisons for HFD groups such as 4 week - M vs F (**Figure 6A**) and 16 week – M vs F (**Figure 6B**) serum proteins. Thereafter we plotted significantly upregulated proteins in these comparisons (**Supplementary Figure 2**). Inflammatory proteins- IL6 and IL17f were upregulated in 4-week male HFD serum samples than female (**Figure 6A**, **Supplementary Figure 2A**). Volcano plots showed muscle and cardiovascular-related proteins – AXIN1 (46) and DDAH1 (47) were upregulated in 16 week male HFD serum samples than female (**Figure 6B**, **Supplementary Figure 2B**). PARP1, involved in DNA repair and cell survival (48) showed significantly higher levels in 16 week male HFD serum samples than female (**Figure 6B**, **Supplementary Figure 2C**).

Olink analysis was also conducted on explant media from 4 week and 6-week HFD male and female GWAT (**Supplementary Tables 3, 5**). We also performed PCA, heatmap (**Supplementary Figures 3**, **4**) and volcano plot analyses (**Supplementary Figure 5**) for GWAT explant proteins. As shown earlier, inflammatory proteins - IL1β and TGFβ1 were significantly higher in 6-week male HFD compared to females (**Figure 3F**, **Supplementary Figure 5**). ENO2 was decreased in 4 week and 6-week male GWAT media (**Supplementary Figures 5A, B**), while CNTN1 was decreased in 4-week male GWAT media (**Supplementary Figure 5A**) compared to females. While, CNTN1 has been shown to be negatively associated with adiposity and obesity (49), ENO2 is a glycolytic enzyme involved in dysregulated adipose function and inflammation (50).

## Discussion

In HFD studies used to investigate obesity, the duration of diet exposure affects systemic and local insulin resistance and inflammation. With obesity, inflammation manifests differently in males and females with profound inflammation in males compared to females, especially in mouse models (14, 38, 39). However, sex differences in impairments during early HFD remain unclear. Our study with 1-, 2-, 4-, 6- and 16-week HFD exhibited sex differences in short term HFD weight gain, insulin resistance along with elevation of inflammatory markers and pro-inflammatory ATMs in the male GWAT. As early as 4 weeks of HFD, serum inflammatory proteins - IL6 and IL17f were higher in males than females. Concurrently, adipocyte hypertrophy increased in male GWAT at short HFD exposure, while adipogenic gene expression in male GWAT was impaired by 16 weeks HFD. These results indicate that male adipocytes show greater impairment in adipocyte hyperplasia as well as metabolic dysfunction compared to females in acute and chronic HFD exposure.

Sex differences in white adipose tissue expansion and inflammation are evident from human and mouse studies (51). Metabolic dysregulation and inflammation in obesity impact susceptibility to cardiovascular diseases and type 2 diabetes (52). Elevated insulin is a common sign of decreased insulin sensitivity, insulin resistance, and a precursor to the development of type 2 diabetes (53). Our studies showed significant sex differences in insulin sensitivity and glucose levels, starting as early as 2 weeks on HFD. HFD male mice presented with elevated insulin levels as compared to HFD female mice. This elevation in glucose and insulin levels was reflected until 6 weeks in HFD male mice. Adipocytes are known to secrete hormones, cytokines, and FFAs, most of which have been shown to play some role in inflammation and systemic insulin resistance (54–56). Concurrently, within 4 weeks of HFD, changes in HFD male serum circulating cytokine concentrations were observed with increased IL6 and IL17f protein levels than females. GWAT explant media also showed elevated levels of IL1β and TGFβ1 at 6 weeks of HFD in males. These cytokines and chemokines potentially contributed to decreased systemic insulin sensitivity in males (57, 58) suggesting induction of adipose tissue dysfunction in males than females with short-term HFD. The evidence supported in this early data lends to the importance of early onset insulin sensitivity impairment as a precursor for inflammation especially in the males (59, 60).

Female mice exhibit lower levels of inflammation and overall metabolic dysfunction when compared to males in the presence of increasing adipose tissue mass (14, 61). Among factors driving such sex differences, sex steroids play a significant biological role in chronic fat inflammation (18, 62). In our study, sexual dimorphism in inflammatory markers developed around 4 weeks. Both *Mcp1* and *Il6* showed increased expression in both short term and long term HFD males than females. Notably, HFD male *Mcp1* and *Il6* expression levels at 4 weeks were higher than at 16 weeks suggesting post-transcriptional regulation of these genes in chronic HFD exposure as observed previously in other studies for *Il6* (39, 43). This data shows that male mice experience increased inflammation early on with HFD compared to their female counterparts. The lower inflammation response in females could be attributed to the protective role of estrogen, which enhanced metabolic function (12, 63). On the other hand, testosterone has been found to impair the regulation of adipose function, potentially causing metabolic dysfunction and higher levels of inflammation in males (53, 64). There is also well documented sexual dimorphism in obesity, where postmenopausal women with declining estrogen tend to have higher rates of obesity and inflammation as compared to men (65, 66).

While markers of inflammation developed around 4 weeks, adipose dysfunction did not appear until a chronic state of obesity. Sex specific responses in fat cell growth were observed with male HFD mice experiencing increased adipocyte hypertrophy that appeared at 4 weeks and continued on throughout HFD exposure. Adipocyte hypertrophy is commonly associated with impaired adipose tissue function, inflammation, and increased free fatty acids eventually leading to systemic hyperlipidemia and liver steatosis (44). At 16 weeks on HFD, male GWAT exhibited significantly lower expression of *Srebp1*, *Cebpa* and *Pgc1a* gene expression compared to male ND mice, suggesting impaired adipogenesis in male HFD mice. Significant differences in *Srebp1* and *Cebpa* expression were not observed in female mice on different diets, highlighting the significance of sex differences in adipogenesis.

Our prior work with lipidomic evaluations highlights pathways that lead to sex differences in lipid metabolism (39, 67). Moreover, sex differences at 4 weeks between the HFD groups emerged in *Lep* and *Pparg*, genes that help regulate insulin sensitivity, glucose tolerance, lipid metabolism, and healthy body weight (28, 39). Lower levels of *Pparg* are associated with chronic obesity, supported by the depleted levels seen in the 16-week HFD males as compared to the ND males. Sex differences in adiponectin gene expression, that helps improve insulin sensitivity (54) did not develop until 16 weeks. HFD male mice presented with significantly depleted levels of *Adipoq* as compared to ND male mice as well as HFD female mice, indicating increased inflammation and impairments in insulin sensitivity (44, 68). This further illustrates sex differences in lipid storage and metabolism between male and female mice, and the significance of sex differences in adipogenesis (28). Long term HFD was also associated with significant protein level changes associated with cellular proliferation, remodeling, lipid metabolism, and inflammation that are hallmarks of obesity. Future studies would benefit from lipidomic evaluations of adipose to understand if the gene expression and adipogenesis differences were a direct result of differing lipid processing. While we specifically assessed changes in adipose tissue and global metabolism, one limitation of this work is that we did not assess liver profiles in 1-, 2- and 4-week HFD states. We know from previous studies from our lab that liver micro-steatosis was observed in male HFD mice at 6 weeks of HFD challenge (14). At 16 weeks, liver steatosis was prominent in male mice on HFD but not females (14).

Our study shows that male HFD mice exhibited a more robust decline in adipogenesis, adipose function, and increase in inflammation as compared to female HFD, highlighting the importance of sex on this timeline and development of adipocyte dysfunction. The timeline of origin of inflammation indicates inflammation occurs prior to the onset of adipose dysfunction and impaired adipogenesis. Therefore, our data suggests that upon onset of HFD, insulin resistance develops first, followed by inflammation, and finally adipose dysfunction becomes significant at a chronic state of obesity. Establishing this timeline allows for a more specific determination of risk factors leading to obesity related co-morbidities and a diminished reliance on BMI as the primary indicator. This timeline also allows for more direct comparisons between metabolic markers and sex. This further highlights the importance of sex in both early and chronic states of obesity and associated metabolic complications (69–71).

Further research is needed to better understand the pathological role of inflammatory and lipid metabolism pathways and their role in increasing susceptibility for obesity and diabetes, considering sex differences. Disease pathogenesis can be monitored by sex alongside the proposed timeline of adipose dysfunction to better understand factors contributing to an increased susceptibility to metabolic dysfunction especially in males and older females. Investigating sex-dependent regulatory pathways can help personalize strategies to treat metabolic disorders.

## Data Availability

The datasets used and/or analyzed during the current study are available from the corresponding author on reasonable request.
