# Supplementary figures and images for: Sex specific pattern of adipose expansion, inflammation and dysfunction with short term high fat diet exposure
Source: Front Endocrinol (Lausanne). 2026 Jun 23;17:1814026. doi: 10.3389/fendo.2026.1814026 (PMC13337368; doi:10.3389/fendo.2026.1814026)

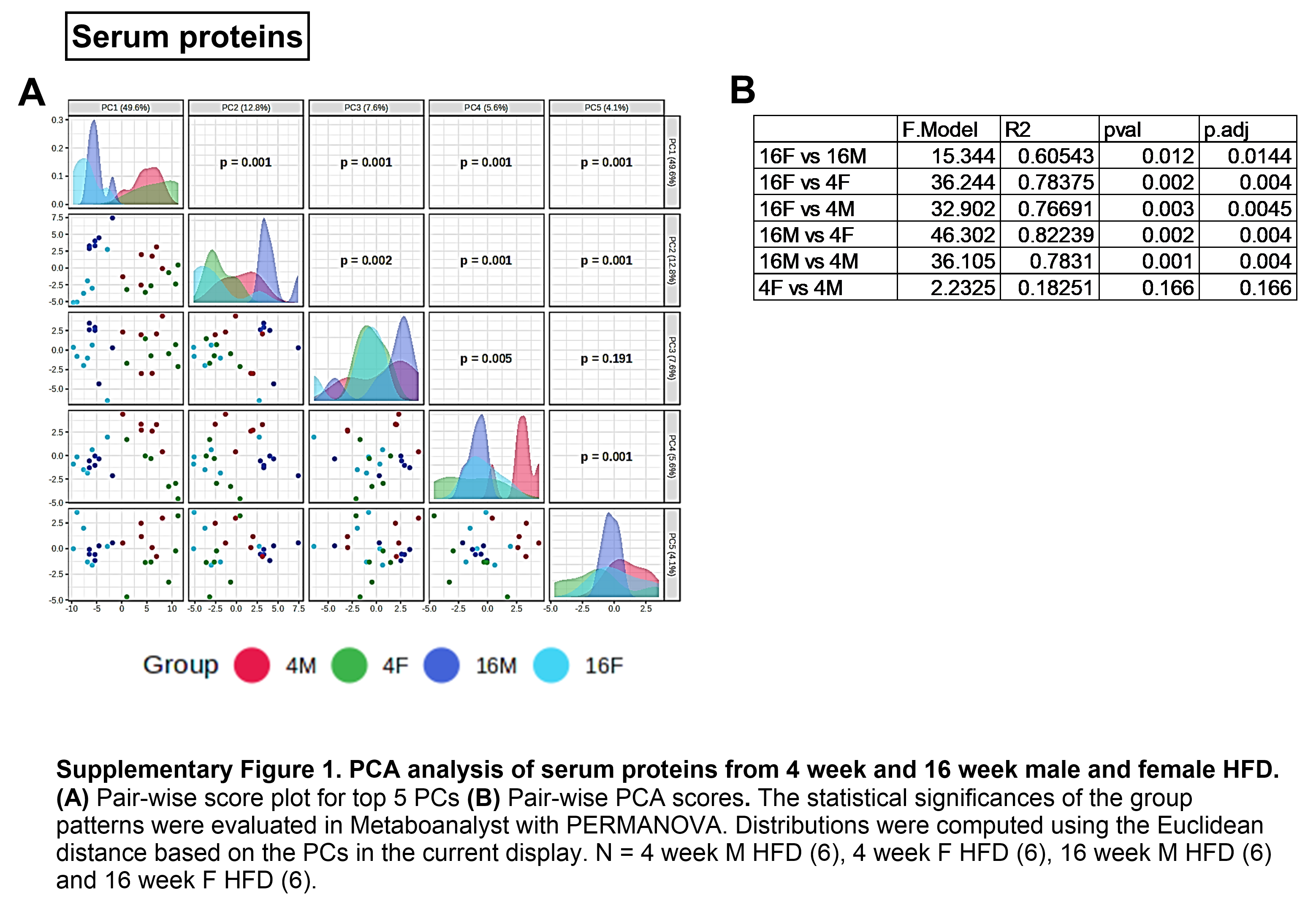

Supplement: Supplementary file 1 [file Image1.tif]

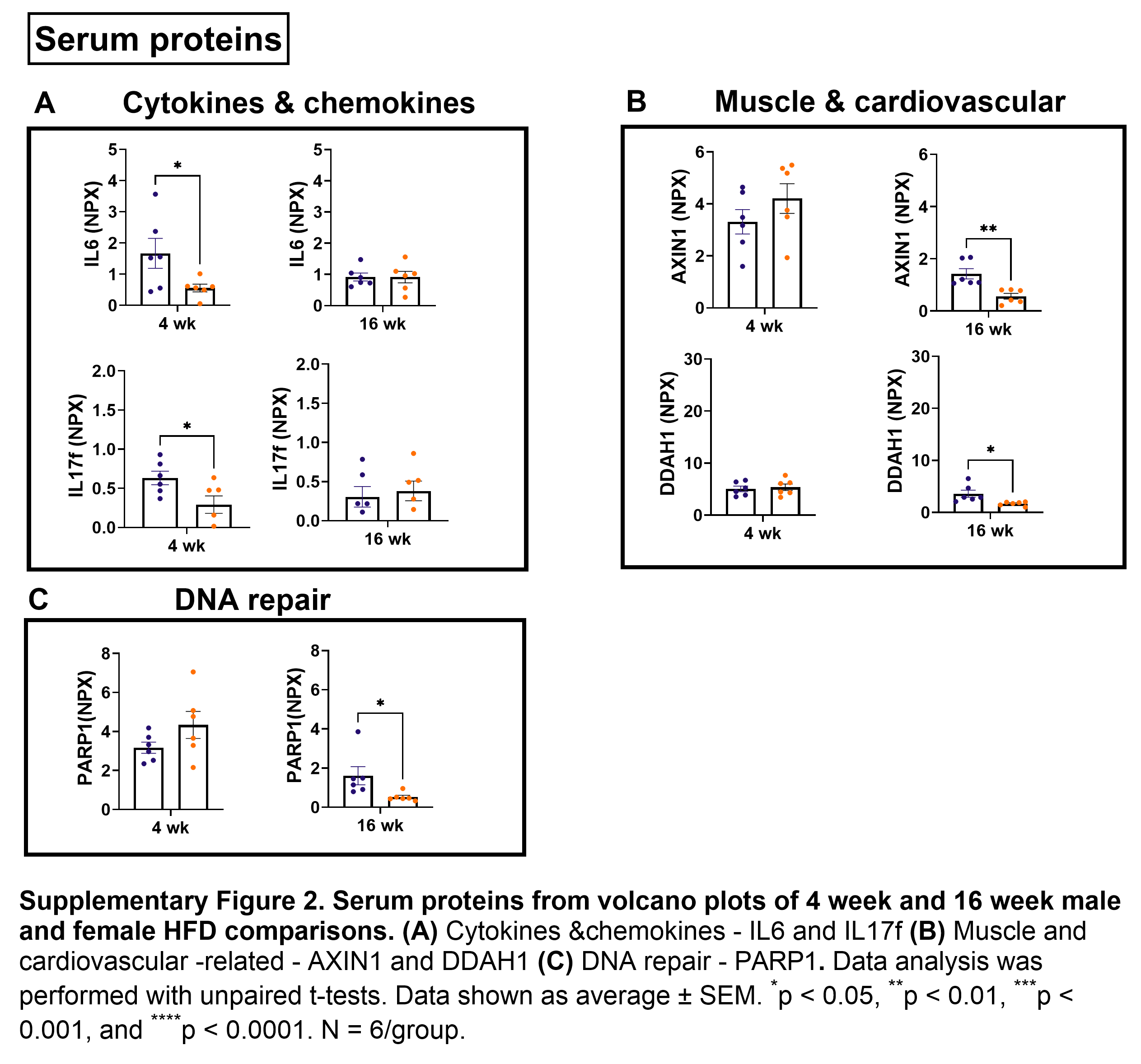

Supplement: Supplementary file 2 [file Image2.tif]

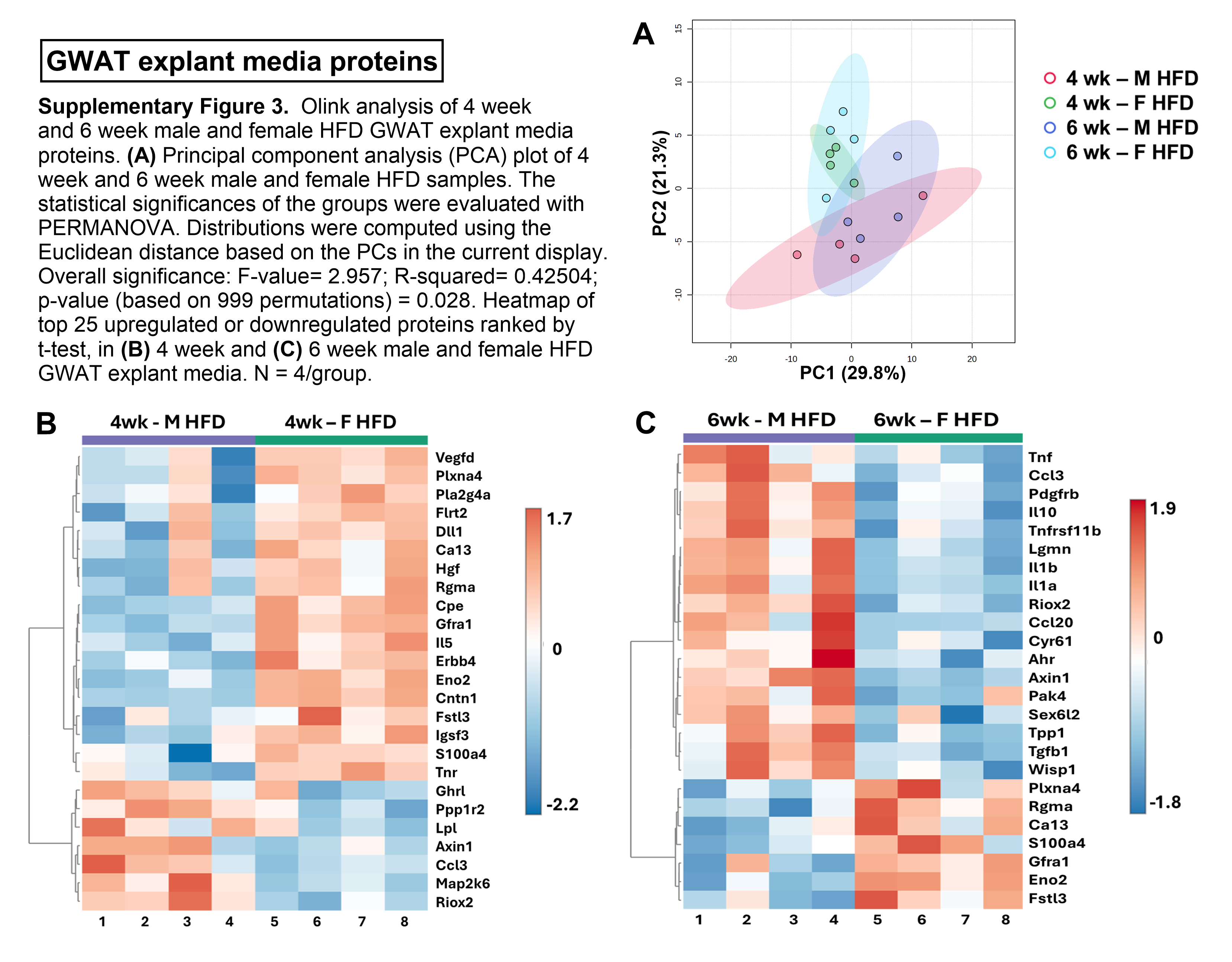

Supplement: Supplementary file 3 [file Image3.tif]

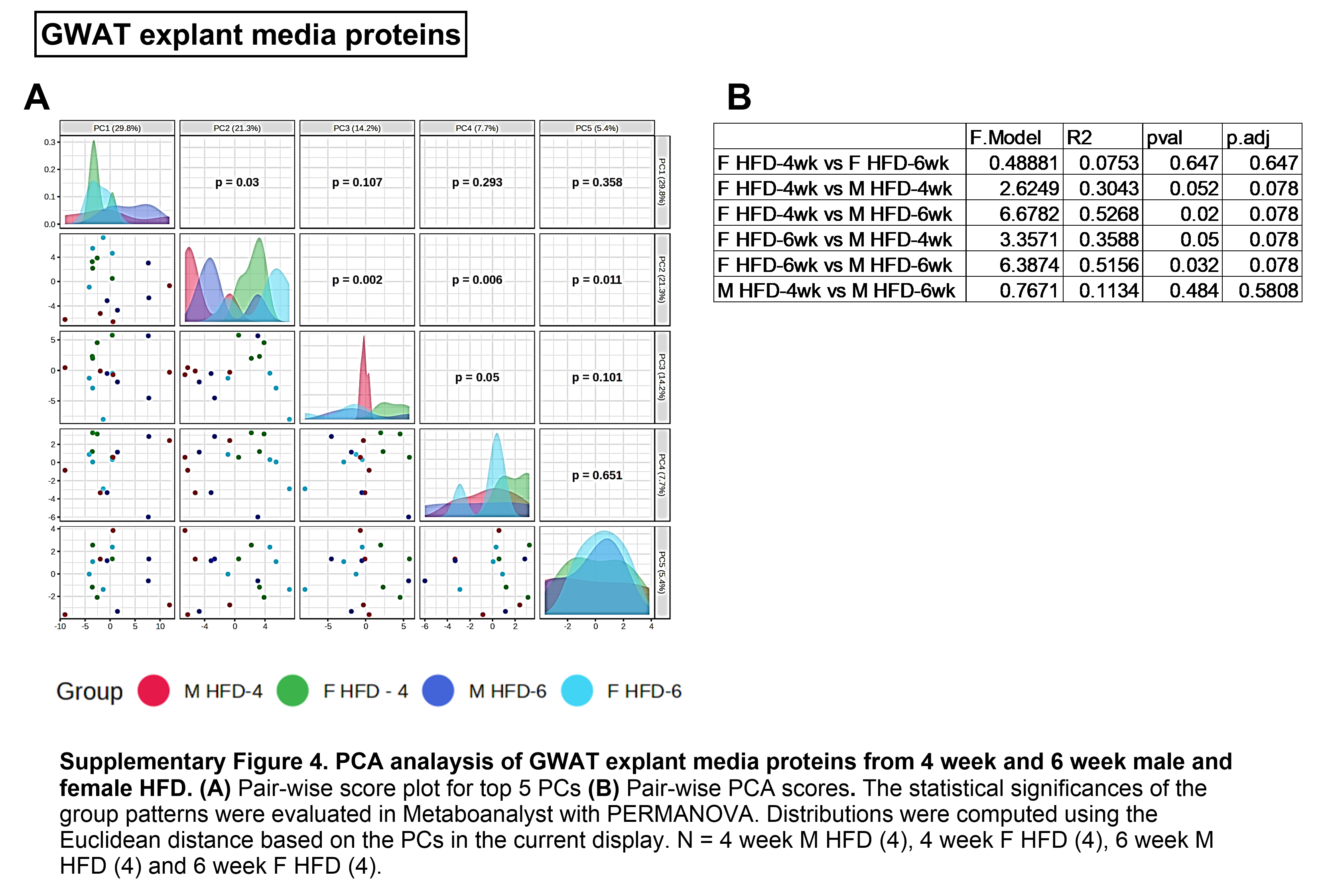

Supplement: Supplementary file 4 [file Image4.tif]

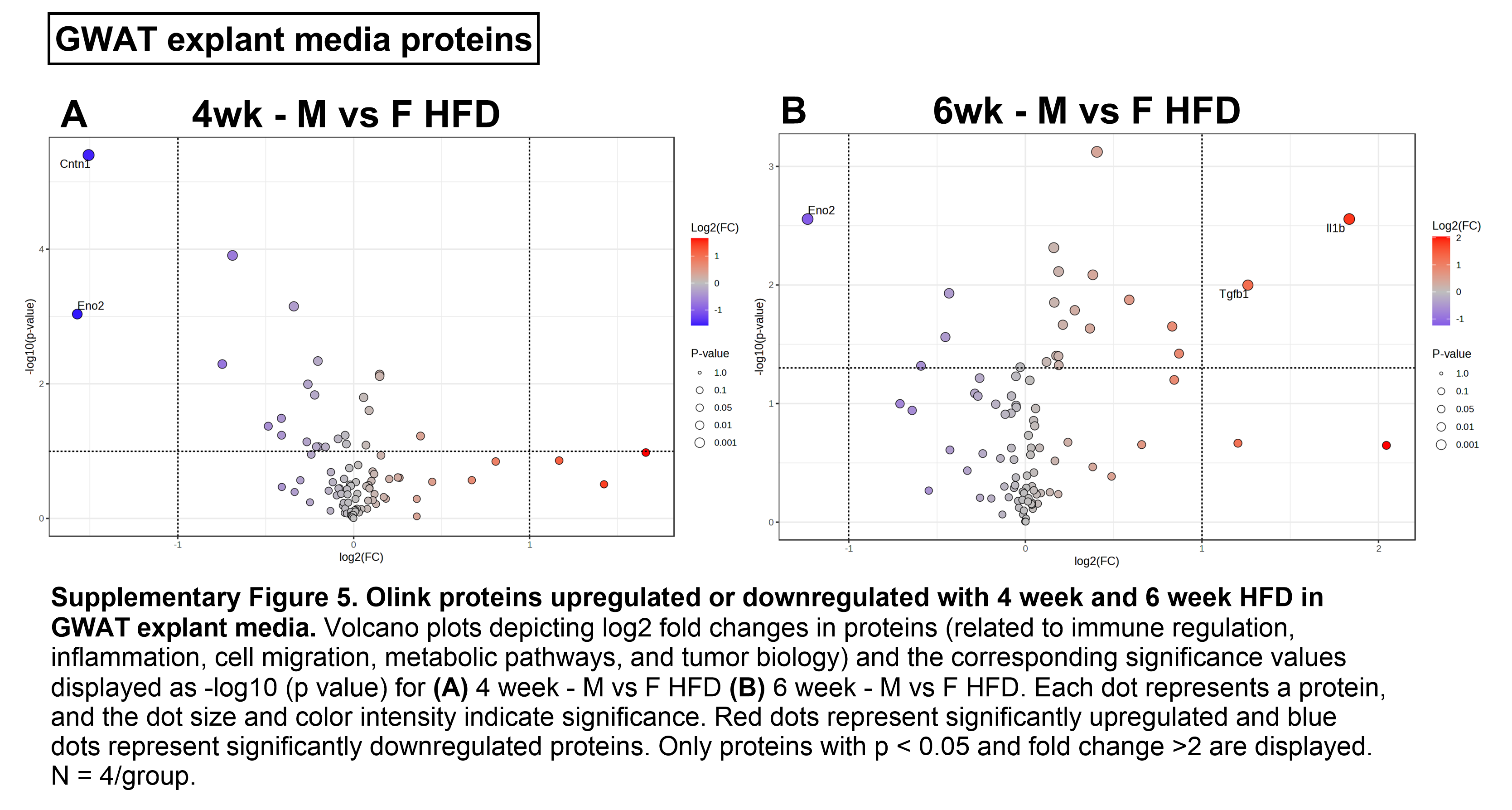

Supplement: Supplementary file 5 [file Image5.tif]
