# Supplementary material for: Sex specific pattern of adipose expansion, inflammation and dysfunction with short term high fat diet exposure
Source: Front Endocrinol (Lausanne). 2026 Jun 23;17:1814026. doi: 10.3389/fendo.2026.1814026 (PMC13337368; doi:10.3389/fendo.2026.1814026)
Supplement: Supplementary file 7 [file Table2.docx]

**Supplementary Table 2. RT-PCR primer sequences**

|  | **Forward Primer (5’ to 3’)** | **Reverse Primer (5’ to 3’)** |
| --- | --- | --- |
| *Arbp* | AGATTCGGGATATGCTGTTGGC | TCGGGTCCTAGACCAGTGTTC |
| *Pgc1α* | TATGGAGTGACATAGAGTGTGCT | CCACTTCAATCCACCCAGAAAG |
| *Pparγ* | GGAAGACCACTCGCATTCCTT | TCGCACTTTGGTATTCTTGGAG |
| *Cebpα* | CAAGAACAGCAACGAGTACCG | GTCACTGGTCAACTCCAGCAC |
| *Srebp1* | GATGTGCGAACTGGACACAG | CATAGGGGGCGTCAAACAG |
| *Mcp1* | TTAAAAACCTGGATCGGAACCAA | GCATTAGCTTCAGATTTACGGGT |
| *Il6* | TAGTCCTTCCTACCCCAATTTCC | AAGGAACCCTTAGAGTGCTTACT |
| *Adipoq* | GCAGGCATCCCAGGACATC | GCGATACATATAAGCGGCTTCT |
| *Leptin* | GAGACCCCTGTGTCGGTTC | CTGCGTGTGTGAAATGTCATTG |
