# Supplementary material for: Sex specific pattern of adipose expansion, inflammation and dysfunction with short term high fat diet exposure
Source: Front Endocrinol (Lausanne). 2026 Jun 23;17:1814026. doi: 10.3389/fendo.2026.1814026 (PMC13337368; doi:10.3389/fendo.2026.1814026)
Supplement: Supplementary file 8 [file Table3.docx]

# Supplementary Table 3. Proteins in the Olink® Target 96 Mouse Exploratory Panel

| **Protein** | **UniProt** | **Protein name** |
| --- | --- | --- |
| Acvrl1 | Q61288 | **Serine/threonine-protein kinase receptor R3** |
| Adam23 | Q9R1V7 | **Disintegrin and metalloproteinase domain-containing protein 23** |
| Ahr | P30561 | **Aryl hydrocarbon receptor** |
| Apbb1ip | Q8R5A3 | **Amyloid beta A4 precursor protein-binding family B member 1-interacting** |
| Axin1 | O35625 | **Axin-1** |
| Ca13 | Q9D6N1 | **Carbonic anhydrase 13** |
| Cant1 | Q8VCF1 | **Soluble calcium-activated nucleotidase 1** |
| Casp3 | P70677 | **Caspase-3** |
| Ccl2 | P10148 | **C-C motif chemokine 2** |
| Ccl20 | O89093 | **C-C motif chemokine 20** |
| Ccl3 | P10855 | **C-C motif chemokine 3** |
| Ccl5 | P30882 | **C-C motif chemokine 5** |
| Cdh6 | P97326 | **Cadherin-6** |
| Clmp | Q8R373 | **CXADR-like membrane protein** |
| Clstn2 | Q9ER65 | **Calsyntenin-2** |
| Cntn1 | P12960 | **Contactin-1** |
| Cntn4 | Q69Z26 | **Contactin-4** |
| Cpe | Q00493 | **Carboxypeptidase E** |
| Crim1 | Q9JLL0 | **Cysteine-rich motor neuron 1 protein** |
| Csf2 | P01587 | **Granulocyte-macrophage colony-stimulating factor** |
| Cxcl1 | P12850 | **C-X-C motif chemokine 1** |
| Cxcl9 | P18340 | **C-X-C motif chemokine 9** |
| Cyr61 | P18406 | **Protein CYR61** |
| Dctn2 | Q99KJ8 | **Dynactin subunit 2** |
| Ddah1 | Q9CWS0 | **N(G),N(G)-dimethylarginine dimethylaminohydrolase 1** |
| Dlk1 | Q09163 | **Delta-like protein 1** |
| Dll1 | Q61483 | **Delta-like protein 1** |
| Eda2r | Q8BX35 | **Tumor necrosis factor receptor superfamily member 27** |
| Eno2 | P17183 | **Gamma-enolase** |
| Epcam | Q99JW5 | **Epithelial cell adhesion molecule** |
| Epo | P07321 | **Erythropoietin** |
| Erbb4 | Q61527 | **Receptor tyrosine-protein kinase erbB-4** |
| Fas | P25446 | **Tumor necrosis factor receptor superfamily member 6** |
| Fli1 | P26323 | **Friend leukemia integration 1 transcription factor** |
| Flrt2 | Q8BLU0 | **Leucine-rich repeat transmembrane protein FLRT2** |
| Foxo1 | Q9R1E0 | **Forkhead box protein O1** |
| Fst | P47931 | **Follistatin** |
| Fstl3 | Q9EQC7 | **Follistatin-related protein 3** |
| Gcg | P55095 | **Pro-glucagon** |
| Gdnf | P48540 | **Glial cell line-derived neurotrophic factor** |
| Gfra1 | P97785 | **GDNF family receptor alpha-1** |
| Ghrl | Q9EQX0 | **Appetite-regulating hormone** |
| Hgf | Q08048 | **Hepatocyte growth factor** |
| Igsf3 | Q6ZQA6 | **Immunoglobulin superfamily member 3** |
| Il10 | P18893 | **Interleukin-10** |
| Il17a | Q62386 | **Interleukin-17A** |
| Il17f | Q7TNI7 | **Interleukin-17F** |
| Il1a | P01582 | **Interleukin-1a** |
| Il1b | P10749 | **Interleukin-1 beta** |
| Il23r | Q8K4B4 | **Interleukin-23 receptor** |
| Il5 | P04401 | **Interleukin-5** |
| Il6 | P08505 | **Interleukin-6** |
| Itgb1bp2 | Q9R000 | **Integrin beta-1-binding protein 2** |
| Itgb6 | Q9Z0T9 | **Integrin beta-6** |
| Kitlg | P20826 | **Kit ligand** |
| Lgmn | O89017 | **Legumain** |
| Lpl | P11152 | **Lipoprotein lipase** |
| Map2k6 | P70236 | **Dual specificity mitogen-activated protein kinase kinase 6** |
| Matn2 | O08746 | **Matrillin 2** |
| Mia | Q61865 | **Melanoma-derived growth regulatory protein** |
| Nadk | P58058 | **NAD kinase** |
| Notch3 | Q61982 | **Neurogenic locus notch homolog protein 3** |
| Ntf3 | P20181 | **Neurotrophin-3** |
| Pak4 | Q8BTW9 | **Serine/threonine-protein kinase PAK 4** |
| Parp1 | P11103 | **Poly [ADP-ribose] polymerase 1** |
| Pdgfb | P31240 | **Platelet-derived growth factor subunit B** |
| Pla2g4a | P47713 | **Cytosolic phospholipase A2** |
| Plin1 | Q8CGN5 | **Perilipin-1** |
| Plxna4 | Q80UG2 | **Plexin-A4** |
| Ppp1r2 | Q9DCL8 | **Protein phosphatase inhibitor 2** |
| Prdx5 | P99029 | **Peroxiredoxin-5, mitochondrial** |
| Qdpr | Q8BVI4 | **Dihydropteridine reductase** |
| Rgma | Q6PCX7 | **Repulsive guidance molecule A** |
| Riox2 | Q8CD15 | **Ribosomal oxygenase 2** |
| S100a4 | P07091 | **Protein S100-A4** |
| Sez6l2 | Q4V9Z5 | **Seizure 6-like protein 2** |
| Snap29 | Q9ERB0 | **Synaptosomal-associated protein 29** |
| Tgfa | P48030 | **Protransforming growth factor alpha** |
| Tgfb1 | P04202 | **Transforming growth factor beta-1 proprotein** |
| Tgfbr3 | O88393 | **Transforming growth factor beta receptor type 3** |
| Tnf | P06804 | **Tumor necrosis factor** |
| Tnfrsf11b | O08712 | **Tumor necrosis factor receptor superfamily member 11B** |
| Tnfrsf12a | Q9CR75 | **Tumor necrosis factor receptor superfamily member 12A** |
| Tnfsf12 | O54907 | **umor necrosis factor ligand superfamily member 12** |
| Tnni3 | P48787 | **Troponin I, cardiac muscle** |
| Tnr | Q8BYI9 | **Tenascin-R** |
| Tpp1 | O89023 | **Tripeptidyl-peptidase 1** |
| Vegfd | P97946 | **Vascular endothelial growth factor D** |
| Vsig2 | Q9Z109 | **V-set and immunoglobulin domain-containing protein 2** |
| Wfikkn2 | Q7TQN3 | **WAP, Kazal, immunoglobulin, Kunitz and NTR domain-containing protein** |
| Wisp1 | O54775 | **WNT1-inducible-signaling pathway protein 1** |
| Yes1 | Q04736 | **Tyrosine-protein kinase Yes** |
